# Revisiting Africa’s Stroke Obstacles and Services (SOS)

**DOI:** 10.1007/s10072-024-07982-y

**Published:** 2025-01-16

**Authors:** Tamer Roushdy, Ahmed Elbassiouny, Selma Kesraoui, Michael Temgoua, Kiatoko Ponte Nono, Selam Kifelew Melkamu, Eitzaz Sadiq, Patty Francis, Oday F. Omar, Waweru Peter, Urvashy Gopaul, Mohammed Faouzi Belahsen, Lukpata Philip Ugbem, Djibrilla Ben-Adji, Noëmie Woodcock, Muhyadin Hassan Mohamed, Sarah Matuja, Chokri Mhiri, Deanna Saylor, Mohamed Maged, Hossam Shokri, Nevine El Nahas

**Affiliations:** 1 Neurology Department, Faculty of Medicine, Ain Shams University, Cairo, Egypt; 2 Department of Neurology, Blida Hospital University, Blida, Algeria; 3 Institute of Applied Neurosciences and Functional Rehabilitation, Bethesda Hospital, Yaoundè, Cameroon; 4 Initiative Plus Hospital Center, Kinshasa, Democratic Republic of the Congo; 5 College of Health Sciences, Department of Neurology, Addis Ababa University, Addis Ababa, Ethiopia; 6 Division of Neurology, Department of Neurosciences, Faculty of Health Sciences, University of Witwatersrand, Johannesburg, South Africa; 7 Neurological Association of South Africa, Randburg, South Africa; 8 Benghazi Medical Center, Benghazi, Libya; 9 Kenyatta University Teaching, Referral & Research Hospital, Nairobi, Kenya; 10 University of Mauritius, Moka, Mauritius; 11 KITE-University Health Network (UHN), Toronto, ON Canada; 12 Neurology Department, Hassan II University Hospital, Sidi Mohamed Ben Abdellah University, Fez, Morocco; 13 Department of Anatomy, Faculty of Basic Medical Sciences, College of Health Sciences, Federal University Wukari, Wukari, Taraba State Nigeria; 14 Centre Hospitalier Régional (CHR) de Maradi, Maradi, Niger; 15 Seychelles Stroke Foundation, Mahe, Seychelles; 16 Department of Neurology, East Africa University Hospital, Bosaso, Somalia; 17 Department of Internal Medicine, Bugando Medical Centre/Catholic University of Health and Allied Sciences, Mwanza, Tanzania; 18 Department of Neurology, Habib Bourguiba University Hospital, Sfax, Tunisia; 19 Department of Medicine, University Teaching Hospital, Lusaka, Zambia; 20 Department of Neurology, School of Medicine, The Johns Hopkins University, Baltimore, MD USA

**Keywords:** Stroke in Africa, Stroke services, Obstacles, Stroke units in Africa, Stroke specialists, Stroke care

## Abstract

**Background:**

As one of the most common non-communicable diseases in Africa, Stroke ought to be dealt with properly with intensifying efforts to control its burden and to face obstacles in its management.

**Methods and Results:**

In this follow-up study we reanalyzed stroke services and related obstacles in 17 African countries that were previously studied in 2021/22 in aspects related to manpower, acute stroke services, rehabilitation programs, number of stroke units/centers, telestroke services, awareness campaigns, and national and international stroke registries through a survey that was sent to stroke specialists and national stroke societies. Overall, there is an improvement in many fields yet many obstacles in the implementation of telestroke services, acute management, secondary prevention, post-discharge services, and follow-ups whether governmental, medical, or societal are prevalent.

**Conclusion:**

Stroke services in Africa are improving in 2024 compared to 2021/22 in many fields, stationary in some fields, and regressing in a few. Managing obstacles that are raised by stroke specialists collectively and on individual countries basis will pave the way for better services for the wellness of stroke victims in Africa.

**Supplementary Information:**

The online version contains supplementary material available at 10.1007/s10072-024-07982-y.

## Introduction

Being a non-communicable disease, stroke is a potentially preventable disease. Yet, for this to be achieved governments ought to progress in solid steps toward establishing proper stroke services and identifying obstacles facing stroke management. They should also follow international guidelines and continuously monitor what is established and what needs further action plans [1].

However, there is a global discrepancy in stroke services between different regions. In Europe, North and South America, Australia, and areas of Asia stroke service is well organized, and action plans are established and properly followed [2–4]. On the other hand, despite the increased burden of stroke in Africa, stroke service is still less defined with deficiencies in many domains [5, 6].

In 2021, Roushdy and colleagues conducted a survey study on 17 countries representing different African regions. They analyzed various aspects of stroke services and concluded that acute stroke care was insufficient regarding the availability of stroke units, reperfusion therapies whether intravenous thrombolysis (IVT) or mechanical thrombectomy (MT), post-stroke rehabilitation, and stroke database registries whether national or international. This study aimed to highlight gaps in stroke service for policymakers [7].

The current study aims to check the status of those gaps and identify whether they were bridged or are still obliterating the progress toward establishing stroke services. Additionally, it is intended to provide a testimonial about obstacles facing stroke service from the point of view of specialists in the field of stroke.

## Methods

This is a follow-up observational comparative descriptive cross-sectional testimonial study conducted on 16 countries that were included in a previous study dating in 2021 [7] – Zanzibar as a portion of Tanzania that was included previously in the 2021 study was merged with the Tanzanian motherland data in the current study – data About Libya was added and compared to the data from the study conducted by Aref and colleagues in 2022 [8] that followed the same parameters of roushdy et al. 2021 study [7].

After receiving ethical approval from the ethical committee of the faculty of medicine – at Ain Shams University, representatives of the 17 countries who were collaborators in the 2021/2022 study were contacted through a questionnaire and were asked to provide their inputs regarding the degree of progression/regression in stroke services besides providing testimonial notes about obstacles facing stroke service in their countries. Country representatives are neurology specialists who have the expertise and are specialized in the field of stroke and who are participating in the implementation of stroke services in their countries through initiatives that are made through regional and continent organizations such as the African Stroke Organization (ASO) or international organizations as the World Stroke Organization (WSO) and its newly implemented program Future Stroke Leaders. The Representatives were further asked to verify their data about stroke from the neurology societies, stroke societies, and ministries of health information discs.

The questionnaire was designed to be a semi-structured availability qualitative questionnaire that aimed at assessing the quality of service under the term whether available or not through Yes/No questions reflecting the potential of service in the designated country with some open-ended questions regarding the manpower as well as the obstacles facing proper implementation of stroke service. It was designed based on the questionnaire that was applied in 2021/2022 studies [7, 8]. It focused mainly on the points related to acute management of stroke as the presence of a sufficient number of neurologists per country, the site where stroke service is provided, as well as rechecking the status of some services that were deficient in 2021/2022 studies as rehabilitation, awareness campaigns, and stroke registries availability.

In 2021/2022 studies, management of stroke risk factors was well organized whether through screening services or availability of medications in different forms. Consequently, in the current questionnaire such domains were not rechecked.

The current questionnaire focused on comparing the availability of parameters of stroke services in 2024 to those previously reported in the study of 2021/2022 which included the following:

1. Manpower: number of neurologists working in the country per 100,000 population.
2. Acute Stroke services: The presence of code stroke, providers of stroke services (General Hospitals, Stroke Units, Stroke Centers), and availability of IVT and MT.
3. Rehabilitation programs through physiotherapy and speech therapy.
4. The total number of stroke units/centers and availability of telestroke services were included.
5. Awareness campaign data and whether in conjunction with international organizations or not and the presence of a national stroke registry database as well as international stroke registry database that was further verified through contacting the Angels, World Stroke Organization (WSO), African Stroke Organization (ASO), the Safe Implementation Treatment for Stroke (SITS international) Karolinska Institute, and the Stroke Care Quality Registry (RESQ) Brno, Czech Republic.

The following terms were defined and highlighted on top of related questions within the questionnaire:

Availability: The fact that something can be used or reached within the country, being a quality measure rather than present in a specific quantity.

Stroke unit: The term stroke unit is meant to reflect the term essential stroke services that are applied in the WSO roadmap for stroke services establishment being a dedicated place for stroke patients with well-equipped 24/7 diagnostic modalities. In our definition of stroke unit and since stroke services in Africa are in their infancy with many steps required for implementation and owing to that nearly all African countries are either of low-income or low-middle income class based on World Bank classification so mandating thrombolysis services to name a dedicated portion in a hospital a stroke unit was skipped.

Stroke Center: The term stroke center is meant to reflect the term advanced stroke services that are applied in the WSO roadmap including the availability of thrombolysis and thrombectomy procedures.

Awareness campaigns: A regular at least annually conducted campaign either along the entire country or along a region of the country that makes use of information services like broadcasting, banners, and audiovisual sources to educate, send a message, and inform the public about specific cause or specific services (stroke as a disorder and ways of managing it whether through primary or secondary measures).

Furthermore, representatives of each country reported obstacles facing proper stroke service in the acute phases, secondary prevention, follow-up, and obstacles facing telestroke service.

For the sake of comparison of services between the years 2021/22 and 2024, data was collected, tabulated in an Excel sheet, presented separately per country as well as collectively into a total of 17 points, and were further presented in frequency and percentages, tables were shaded in green for stationary (yes) or progression (yes with an up arrow), beige for stationary (no) or regression (no with a down arrow), and collective stationary categories were presented with a horizontal two-directional arrow.

## Results

The population of the 17 participating African countries increased approximately by 61 million within 3 years. Nine countries had an increase in the number of working neurologists, the number did not change in 2 countries and decreased in 5 other countries.

The number of neurologists per 100,000 population increased in 5 countries, showed no change in 2, and decreased in 9 countries (Table 1).

**Table 1:**
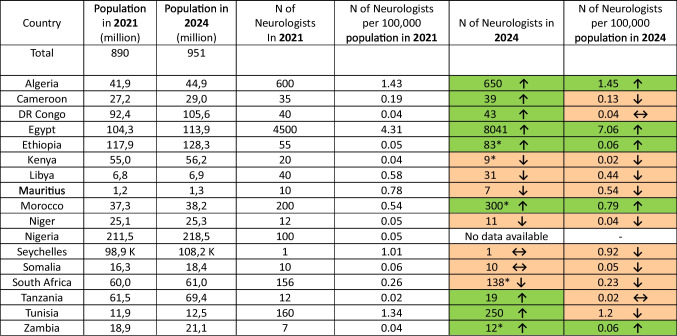
Country population and working neurologists

K: thousands

* Neurologists in Ethiopia are located within major cities and serve in 17 out of the 335 hospitals, Neurologists in Kenya serve in urban referral centers only,

In addition to the 300 neurologists in Morocco, there are 100 under training,

South Africa neurologists are divided into 101 in the private sector, 37 in public hospitals, and 27 under training,

9 of the 12 neurologists in Zambia are based in the capital city of Lusaka.

Code Stroke did not differ between 2021/22 and 2024, while Stroke units became available in 13 (76.5%) countries compared to 5 (29.41%) in 2021.

**Fig. 1:**
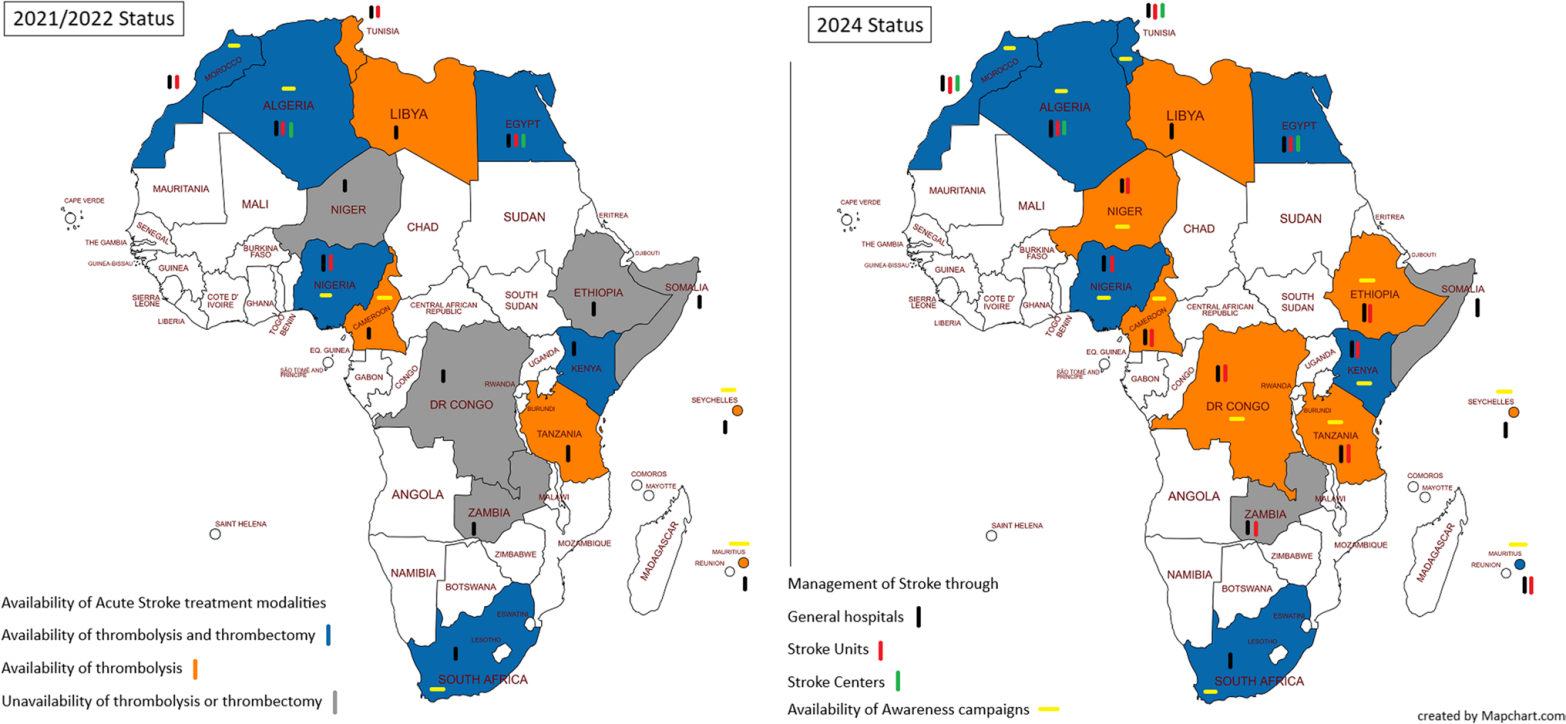
An Infographic map of the changes that took place in stroke services between 2021/22 and 2024

As for Stroke centers, they became available in 2 additional countries besides the original 2 countries, with an increase from (11.8%) to (23.5%). Countries utilizing IVT increased from 12 countries (70.6%) in 2021/22 to 15 (88.2%) in 2024, and MT from 6 (35.3%) in 2021/22 to 8 countries (47.1%) (Table 2, Fig. 1).

**Table 2:**
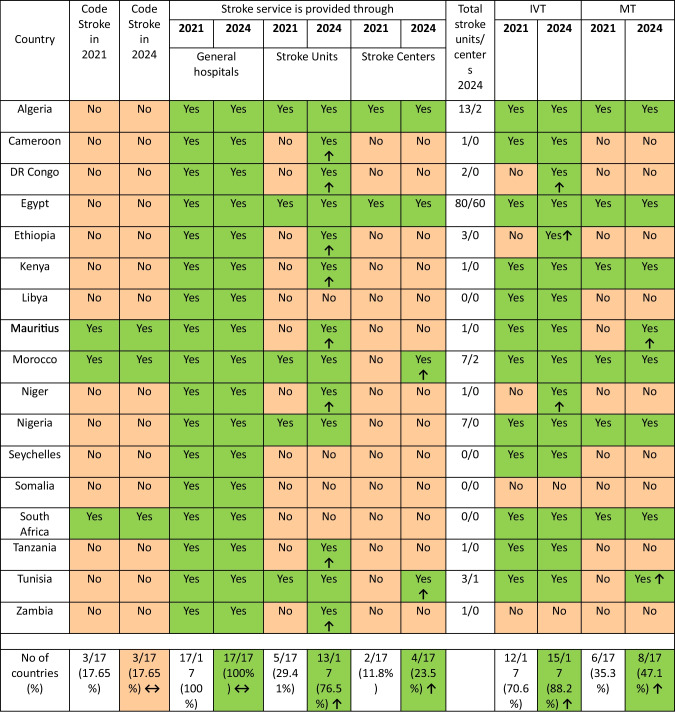
Acute phase management

IVT: Intravenous thrombolysis, MT: Mechanical thrombectomy

Ethiopia was offered IVT through an initiative from WSO in a project sponsored by WSO’s future stroke leaders’ program.

In 2024, physiotherapy is still collectively available in 13 (76.5%) countries with the introduction of the service in Tanzania yet providing the service to stroke patients in Ethiopia has shifted to serve trauma due to the civil unrest.

As for speech therapy, it progressed from being available in 10 countries (58.8%) to becoming available in 11 (64.7%). Stroke awareness campaigns showed a substantial increase from being present in 7 (41.2%) countries in 2021/22 to 13 (76.5%) countries in 2024, attributable to collaboration with international stroke organizations (the Angels, WSO, and ASO) in 9 out of the 13 countries (Fig. 1). Despite that telestroke service was not analyzed in 2021 yet, in the current study it was found to be present in 4 countries, with an ongoing connection between Egypt and Somalia in this context. The availability of national Stroke registry was introduced in Algeria to be available in a total of 4 (23.5%) countries and is under construction in Ethiopia and Mauritius. As for engagement in international stroke registries whether the SITS or RESQ it was introduced in Nigeria and Tunisia and dropped from Kenya (Table 3).

**Table 3:**
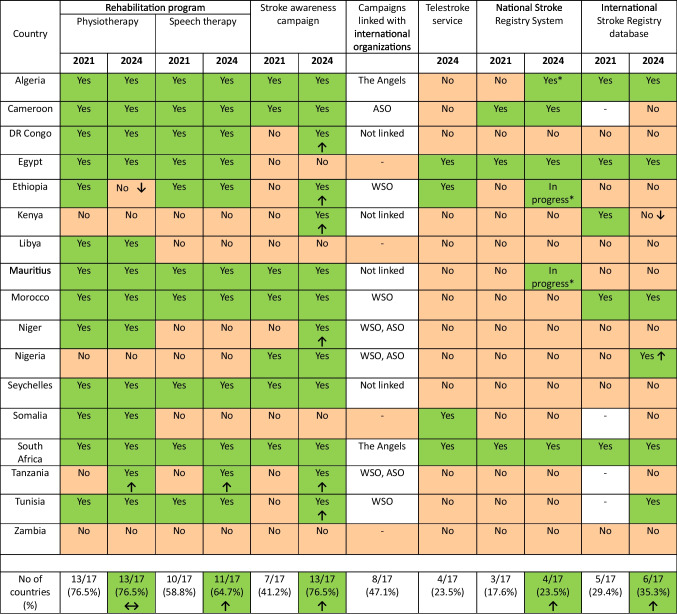
Post-discharge, Tele services, and quality registries

ASO: African Stroke Organization (Africa Day of Stroke), WSO: World Stroke Organization (World Day of Stroke)

*In Algeria there is an established regional stroke registry in the county of Blida (1.5 million inhabitants), while in Ethiopia and Mauritius, a national stroke registry is currently under construction.

Obstacles facing the implementation of telestroke services, acute management, secondary prevention, post-discharge services, and follow-ups were present in all 17 countries with varying percentages and reasons. There was nearly a constant ratio preserved between the 5 North African countries and the 12 Sub-Saharan countries. Governmental reasons represented major obstacles related to telestroke service implementation. Societal factors contributed to acute stroke care and secondary prevention, post-discharge, and follow-ups. Meanwhile, medical reasons were commonly encountered in acute stroke care (Table 4) (supplementary Table 1).

**Table 4:** Obstacles facing Stroke Services in African Countries based on testimonials of neurologists and stroke specialists

		Telestroke service		Hyperacute and acute stroke care		Secondary prevention, post-discharge, and follow-up	
		Freq (%)	Names	Freq (%)	Names	Freq (%)	Names
Governmental	Lack of organized infrastructure communication	11/17 (64.7%) NA: 3/5 (60%) SSA: 8/12 (66.6%)	DZ, CM, DRC, LY, MU, MAR, NER, NGR, SYC, ZA, TZA	-		-	
	Not priority	4/17 (23.5%) NA: 1/5 (20%) SSA: 3/12 (25%)	DZ, DRC, KE, MU	3/17 (17.6%) NA: 0% SSA: 3/12 (25%)	DRC, NER, SOM	1/17 (5.9%) NA: 0% SSA: 1/12 (8.3%)	SYC
	No implementation plan	3/17 (17.6%) NA: 1/5 (20%) SSA: 2/12 (16.6%)	MAR, NGR, SYC	-		-	
	Lack of funding and high cost	3/17 (17.6%) NA: 0% SSA: 3/12 (25%)	KE, MU, ZA	2/17 (11.8%) NA: 1/5 (20%) SSA: 1/12 (8.3%)	TUN, ZMB	1/17 (5.9%) NA: 0% SSA: 1/12 (8.3%)	ZA
Societal	Lack of legal framework to clarify the responsibility	1/17 (5.9%) NA: 1/5 (20%) SSA: 0%	TUN	-		-	
	Investigations are not uploaded to the system	1/17 (5.9%) NA: 0% SSA: 1/12 (8.3%)	ET	-		-	
	Lack of awareness	1/17 (5.9%) NA: 1/5 (20%) SSA: 0%	LY	11/17 (64.7%) NA: 3/5 (60%) SSA: 8/12 (66.6%)	EG, KE, MU, MAR, NGR, SYC, SOM, ZA, TZA, TUN, ZMB	11/17 (64.7%) NA: 4/5 (80%) SSA: 7/12 (58.3%)	DZ, EG, KE, LY, MU, NER, SOM, ZA, TZA, TUN, ZMB
	Financial burden	-		4/17 (23.5%) NA: 0% SSA: 4/12 (33.3%)	DRC, KE, MU, SOM	6/17 (35.3%) NA: 0% SSA: 6/12 (50%)	CM, DRC, ET, KE, ZA, ZMB
Medical	No emergency stroke medical services or system	-		9/17 (53%) NA: 3/5 (60%) SSA: 6/12 (50%)	CM, EG, MU, MAR, NGR, ZA, TUN, ZMB, CM	-	
	Shortness in stroke units, centers, and thrombectomy	-		10/17 (58.8%) NA: 3/5 (60%) SSA: 7/12 (58.3%)	DZ, CM, DRC, ET, KE, LY, MAR, NER, SYC, TZA	-	
	Lack of neuroimaging	1/17 (5.9%) NA: 0% SSA: 1/12 (8.3%)	ZMB	1/17 (5.9%) NA: 0% SSA: 1/12 (8.3%)	ET	-	
	A limited number of neurologists/neuro interventionists or training	2/17 (11.8%) NA: 0% SSA: 2/12 (16.6%)	ZA, ZMB	8/17 (47.1%) NA: 2/5 (40%) SSA: 6/12 (50%)	CM, ET, KE, LY, MU, MAR, NER, ZA	2/17 (11.8%) NA: 0% SSA: 2/12 (16.6%)	SYC, SOM
	Reduced awareness and experience	3/17 (17.6%) NA: 1/5 (20%) SSA: 2/12 (16.6%)	DZ, ET, MU	-		-	
	Lack of management plan	-		-		6/17 (35.3%) NA: 3/5 (60%) SSA: 3/12 (25%)	LY, MU, MAR, NGR, TUN, ZMB

DZ: Algeria, CM: Cameroon, DRC: Democratic Republic of Congo, LY: Libya, MU: Mauritius, MAR: Morocco, NER: Niger, NGR: Nigeria, SYC: Seychelles, ZA: South Africa, TZA: Tanzania, KE: Kenya, SOM: Somalia, ZMB: Zambia, TUN: Tunisia, ET: Ethiopia, EG: Egypt, NA: North Africa, SSA: Sub Saharan Africa

## Discussion

Stroke is one of the most common neurological disorders worldwide with direct and indirect economic consequences [9].

Its burden has declined in high-income countries (HICs) owing to placing it as a top priority with continuous improvements in care provided to patients in addition to continuous research in the field of stroke and adequate funding [10].

On the contrary, the incidence of stroke has increased in LMIC, given that nearly all African countries belong to LMIC except for Seychelles which despite belonging to HIC still has a shortage of services [11]. This shortage reflects that the economic status of countries is not the only factor that could hinder stroke services, but it is the arrangement of priorities among governments. Stroke in Africa needs to be prioritized especially that the burden is rising owing to many factors, such as longevity, hypertension, diabetes, obesity, and sedentary lifestyle, in addition, shifting from rural to urban areas is associated with stress. All such factors ought to push governments and non-governmental organizations to prioritize stroke care [12].

African countries can make use of the WSO roadmap that divides stroke care from minimal services, through essential services, up to advanced services. So, governments could check the current stroke service availabilities in their countries and follow the roadmap [13].

Stroke services are improving in Latin America owing to joining efforts between governments and international organizations in the field of stroke; African countries might make use of the process that took place in Brazil and in different Latin American countries and approach stroke in a similar manner [4, 14].

Continuous basic and advanced research in the field of stroke is much needed in Africa knowing that the incidence of stroke is as high as 316 per 100,000, the prevalence sums up to 1460 per 100,000 and fatality within 3 years is around 84% [15].

The current study found that the African population is continuously growing with more than 60 million increases within the last 3 years. Despite that more than half of the participating countries had an increase in the working neurologists yet still not mounting to the minimal World Health Organization (WHO) recommendations of at least 1 per 100,000 persons in most countries [16]. Only Algeria, Egypt, and Tunisia have more than 1 neurologist per 100,000, and only Egypt has 7.06 in 2024, thus exceeding the European estimates of 4.84 neurologists per 100,000 [17].

Such deficiency in manpower is one of the obstacles facing stroke service in Africa moreover, the available neurologists are unevenly distributed in some countries between hospitals and between urban and rural areas as found in Ethiopia, Kenya, South Africa, and Zambia.

Facing manpower deficiency is approachable through different aspects and some of these aspects were tried in some African countries and have proven efficacy. In Nigeria task shifting and task sharing as an approach to deal with non-communicable diseases and face deficiencies in some medical specialties as neurology was made use of by the federal ministry of health [18].

Other ways of dealing with manpower deficiencies include partnerships between ministries of health in developing countries and developed ones. There are also faculties as the Wessex-Ghana Stroke partnership to train neurologists who in turn train their deputies and continue the cycle of training and auditing under the term train the trainer [19].

Implementing a training residency program where young neurologists can be trained through importing expertise to developing countries as the program implemented in Ethiopia could be tried in different African countries [20]. However, we need to tailor the programs of developed countries to fit in developing ones taking into consideration the deficiency in resources [21].

In the acute phase of stroke management, it was found that the number of countries with stroke units increased, where 13 countries have a total of 121 stroke units and 4 countries have 65 stroke centers.

Although Egypt has the maximum number of 80 units and 60 centers yet, this number is still defective given that Egypt has an annual incidence of 150,000–250,000 stroke cases and thus needs approximately 500 units [22]. Also, this number seems much lower than that reported by De Sousa and colleagues of 2165 stroke units in 44 European countries in 2019 [23].

According to testimonials from representatives from sub-Saharan Africa as well as from North Africa, deficiency in stroke units and centers was considered a major obstacle to acute stroke care in 58.8% of countries.

African countries might make use of ongoing initiatives from international organizations such as the WSO to implement stroke services including building expertise to take over the management of stroke units and be a nidus for replication. This should take into consideration the equality in distributing stroke services in underrepresented areas as rural regions, borders, and governorates away from capitals [24, 25].

Meanwhile, patients in their communities are to be approached either by stroke specialists or through training general practitioners and primary care providers to triage patients and refer those in need of acute management to central hospitals or available stroke units and centers. This approach might facilitate accessibility to services for the majority of the population until stroke services are implemented in a proper geographical representation. Adapting the “use of map stroke”, that was applied in Brazil and took into consideration the population density, the burden of stroke, and the needed stroke units and its catchment area, might be of benefit in Africa [26].

Acute phase intervention whether through IVT or MT has progressed in 2024 compared to 2021 with the introduction of three and two additional countries for IVT and MT respectively. Nevertheless, this is still insufficient taking into account that both managements are cornerstones in acute stroke care [22]. Still, Somalia and Zambia are deficient in both, and Cameron, Democratic Republic of Congo, Ethiopia, Libya, Niger, Seychelles, and Tanzania are deficient in MT.

Deficiencies in IVT and MT have two facets. The first one is the under-established infrastructure from angio-suites to availability of materials for MT and vials for IVT. This needs campaigns targeting policymakers and stakeholders with data showing that the financial burden from disability arising from stroke exceeds the budget needed for revascularization. Such a campaign was initiated in Egypt and could persuade the government with the importance of endorsing both IVT and MT [9, 27].

The second facet is the deficiency in expertise, and this could be solved by training and hands-on workshops that can improve hand skills and reduce door to groin as was shown by Inoa and colleagues [28].

Code Stroke showed a stationary status in 2024 compared to 2021 being available only in 3 countries. This was attributed to the lack of emergency stroke medical services in 9 countries and to stroke being non-priority for governments in 3 countries. Code Stroke is an item within any acute stroke service management so once more stroke units and centers are implemented; code stroke will be in turn applied within the stroke pathway of such units and centers.

Despite rehabilitation services availability maintained its status in the 2024 analysis with a total of 13 countries. Physiotherapy showed a true decline in Ethiopia after the diversion of services to victims of violence due to civil unrest. Physiotherapy and speech therapy were introduced in Tanzania in 2024 compared to 2021, yet still, 4 countries lack physiotherapy, and 6 countries don’t provide speech therapy owing to being a non-priority, lack of funding, and high cost of service in addition to a lack of awareness of its importance.

In contradistinction to 2021, Tunisia started to become active in the SITS database in 2024. Nigeria began to participate in RESQ according to confirmation from Brno, Czech Republic RESQ headquarters. Mauritius initiated a National Stroke Registry through local governmental initiatives and Ethiopia through cooperation between the government and the WSO and its future stroke leaders’ initiatives, while Kenya no longer became active in the SITS database.

All countries became active in awareness campaigns except for Egypt, Libya, Somalia, and Zambia [29]. This came through engagement of many countries with international organizations either the Angels, the Stroke Awareness Day of the ASO, WSO, or both [12].

Despite the introduction of awareness campaigns, still social awareness is lacking in 11 countries which negatively impacts acute management since patients fail to arrive at hospitals within the window. It similarly impacted secondary prevention strategies, post-discharge, and compliance regarding follow-ups. Governmental and non-governmental engagement in awareness campaigns using information services like broadcasting, banners, and audiovisual sources to educate the entire population about stroke management whether in the acute or post-acute phase will reduce stroke burden on a long-term basis.

Finally, it was found that telestroke services are present in only four countries owing to governmental nonpriority, poor communication infrastructure, and high implementation costs.

The current study has points of power as it succeeded in involving the same countries included in the 2021/22 studies. It also could confirm data accuracy through verification from neutral sources as is the case in international database quality registries. This study throws light on obstacles facing stroke services from a testimonial perspective of those incorporated in managing stroke in their countries and through different national stroke societies. Meanwhile, the current study has some limitations, being a comparative study to that held in 2021/22 made it difficult to include new countries and in turn obstacles facing stroke services could not be generalizable to all African countries. The current study failed to analyze the total number of patients receiving IVT or MT per country and only mentioned availability. Within the current study management of stroke risk factors and primary management was not analyzed as screening programs and availability of medications for various vascular risk factors of stroke were optimum in 2021/22 studies. Despite being a study that analyzes stroke service in one continent, Africa still has some variabilities in its countries such as those related to the economic and socio-political status that might have a positive or negative influence on stroke services and encountered obstacles so future studies approaching stroke services based on socio-political and health-economics is recommended.

## Conclusion

Stroke services in Africa are improving in 2024 compared to 2021/22 in many fields, stationary in some fields and regressing in a few. Managing obstacles that are raised by stroke specialists collectively and on individual countries basis will pave the way for better services for the wellness of stroke victims in Africa.

## Supplementary Information

Below is the link to the electronic supplementary material.

Supplementary file1 (DOCX 22 KB)

## Data Availability

Full data is available by the corresponding author on asking based on reasonable causes.

## Abbreviations

IVT: Intravenous thrombolysis
MT: Mechanical thrombectomy
WSO: World Stroke Organization
ASO: African Stroke Organization
SITS: Safe Implementation Treatments for Stroke
RESQ: Registry Care Stroke Quality
HICs: High-Income Countries
LICs: Low-Income Countries
LMICs: Low Middle-Income Countries
WHO: World Health Organization

## References

[1] Owolabi MO, Thrift AG, Mahal A, Ishida M, Martins S, Johnson WD et al (2022) Primary stroke prevention worldwide: translating evidence into action. Lancet Public Health 7(1):e74–e85. 10.1016/S2468-2667(21)00230-9 34756176 10.1016/S2468-2667(21)00230-9 PMC8727355

[2] Ouriques Martins SC, Sacks C, Hacke W, Brainin M, de Assis Figueiredo F, Marques Pontes-Neto O et al (2019) Priorities to reduce the burden of stroke in Latin American countries. Lancet Neurol. 18(7):674–683. 10.1016/S1474-4422(19)30068-7 31029579 10.1016/S1474-4422(19)30068-7

[3] The Lancet Neurology (2020) A unified European action plan on stroke. Lancet Neurol 19(12):963 33181090 10.1016/S1474-4422(20)30409-9

[4] Martins SCO, Lavados P, Secchi TL, Brainin M, Ameriso S, Gongora-Rivera F et al (2021) Fighting against Stroke in Latin America: a joint effort of medical professional societies and governments. Front Neurol 12:743732 34659101 10.3389/fneur.2021.743732 PMC8517273

[5] Sarfo FS, Akpa OM, Ovbiagele B, Akpalu A, Wahab K, Obiako R et al (2023) Patient-level and system-level determinants of stroke fatality across 16 large hospitals in Ghana and Nigeria: a prospective cohort study. Lancet Glob Health 11(4):575–585 10.1016/S2214-109X(23)00038-4 PMC10080070 36805867

[6] Walker R (2022) Osuntokun Award Lecture 2021: Challenges of Measuring the Burden of Stroke in Africa. J Stroke Cerebrovasc Dis 31(4):106386 35317913 10.1016/j.jstrokecerebrovasdis.2022.106386

[7] Roushdy T, Aref H, Kesraoui S, Temgoua M, Nono KP, Gebrewold MA et al (2022) Stroke services in Africa: What is there and what is needed. Int J Stroke 17(9):972–982 35034522 10.1177/17474930211066416

[8] Aref H, El Nahas N, Alrukn SA, Khan M, Kesraoui S, Alnidawi F et al (2023) Stroke services in MENA: What is there and what is needed. PLoS ONE 18(7):e0288030 37471350 10.1371/journal.pone.0288030 PMC10358887

[9] Aref H, El Nahas N, Elsisi GH, Shokri H, Roushdy T (2023) The budget impact of alteplase in the treatment of acute ischemic stroke in Egypt. Front Neurol 14:1220615 38020606 10.3389/fneur.2023.1220615 PMC10663356

[10] Kiiza MC, Wanzhu Z (2023) Stroke fatality in sub-Saharan Africa: time for action. Lancet Glob Health 11(4):489–490 10.1016/S2214-109X(23)00081-5 36805868

[11] Banerjee TK (2022) Editorial: Neuroepidemiology of stroke in low and middle income countries. Front Neurol 13:1059974 36425803 10.3389/fneur.2022.1059974 PMC9680526

[12] Akinyemi RO, Ovbiagele B, Adeniji OA, Sarfo FS, Abd-Allah F, Adoukonou T, Ogah OS, Naidoo P, Damasceno A, Walker RW, Ogunniyi A, Kalaria RN, Owolabi MO (2021) Stroke in Africa: profile, progress, prospects and priorities. Nat Rev Neurol 17(10):634–656 34526674 10.1038/s41582-021-00542-4 PMC8441961

[13] Lindsay P, Furie KL, Davis SM, Donnan GA, Norrving B (2014) World stroke organization global stroke services guidelines and action plan. Int J Stroke 9(SA100):4–13 25250836 10.1111/ijs.12371

[14] Silva GS, Rocha ECA, Pontes-Neto OM, Martins SO (2018) Stroke care services in Brazil. J Stroke Med 1(1):51–54

[15] Okekunle AP, Jones S, Adeniji O, Watkins C, Hackett M, Di Tanna GL et al (2023) Stroke in Africa: a systematic review and meta-analysis of the incidence and case-fatality rates. Int J Stroke 18(6):634–644 36503371 10.1177/17474930221147164 PMC10313746

[16] Santos-Lobato BL, Tomaselli PJ, Santos-Lobato EAV, Cassenote AJF, Cabeça HLS (2023) There is no shortage, but inequality: demographic evolution of neurologists in Brazil (2010–2020). Arq Neuropsiquiatr 81(2):134–145 36948199 10.1055/s-0043-1761490 PMC10033185

[17] Kissani N, Liqali L, Hakimi K, Mugumbate J, Daniel GM, Ibrahim EAA et al (2022) Why does Africa have the lowest number of Neurologists and how to cover the Gap? J Neurol Sci 434:120119 34982975 10.1016/j.jns.2021.120119

[18] The Federal Ministry of Health (2021) Policy on Task-Shifting and Task-Sharing for the control of non-communicable disease in Nigeria. An Addendum to the Federal Ministry of Health (FMOH) Task-Shifting / Task-Sharing Policy For Essential Health Care Services in Nigeria (2018)

[19] Johnson L, Akpalub A, Ananeb D, Cudjoeb C, Easton S, Laryeab R et al (2017) Multi-disciplinary stroke care in developing countries – lessons from the Wessex-Ghana stroke partnership. South Sudan Med J 10(4):84–86

[20] Belay HD, Gebrewold MA, Ayele BA, Oda DM, Kelemu FT, Zewde YZ et al (2024) Neurology training and medical education in resource-limited settings: building and growing the first neurology residency program in East Africa. Semin Neurol 44(2):147–158 38631360 10.1055/s-0044-1785539

[21] Habibi J, Bosch J, Bidulka P, Belson S, DePaul V, Gandhi D et al (2023) Strategies for specialty training of healthcare professionals in low-resource settings: a systematic review on evidence from stroke care. BMC Med Educ 23(1):442 37328888 10.1186/s12909-023-04431-w PMC10273731

[22] Hacke W, Caso V, Esagunde RU, Aref H, Martins SCO, Mikulik R (2021) Stroke care taking flight with the wings of ANGELS. A symposium presented by Boehringer Ingelheim at the ESOWSO Virtual Conference 08 November 2020. CNS 6:8–25

[23] Aguiar de Sousa D, Wilkie A, Norrving B, Macey C, Bassetti C, Tiu C et al (2023) Delivery of acute ischaemic stroke treatments in the European region in 2019 and 2020. Eur Stroke J 8(3):618–628 37431768 10.1177/23969873231186042 PMC10472963

[24] Martins SCO, Secchi TL, Molina C, Nogueira R (2023) Editorial: Development of stroke systems of care across the globe. Front Neurol 14:1292036 37830086 10.3389/fneur.2023.1292036 PMC10565845

[25] Roushdy T, Alet MJ, Lotlikar R, Ramage E, Ullberg T, Mosconi MG et al (2023) Applying the World Stroke Organization roadmap in planning a model for stroke service implementation in Matrouh Governorate-Egypt: a World Stroke Organization young future stroke leaders’ analytical study. Egypt J Neurol Psychiatry Neurosurg 59:150

[26] Carbonera LA, Rivillas JA, Gordon Perue G, da Luz DL, Boiani M, de Souza AC et al (2024) The MAPSTROKE project: a computational strategy to improve access to acute stroke care. Int J Stroke 19(7):747–753 38346937 10.1177/17474930241234528

[27] Zakaria MF, Aref H, AbdElNasser A, Fahmy N, Tork MA, Fouad MM et al (2018) Egyptian experience in increasing utilization of reperfusion therapies in acute ischemic stroke. Int J Stroke 13(5):525–529 28585904 10.1177/1747493017711949

[28] Inoa V, Then R, Cancelliere NM, Spiegel GR, Fraser JF, Hepburn M et al (2024) Mechanical thrombectomy workshops improve procedural knowledge and skills among neurointerventional teams in low- to middle-income countries. Stroke 55(7):1886–1894 38913795 10.1161/STROKEAHA.124.046516

[29] Akinyemi RO, Brainin M (2021) The African Stroke Organization - a new dawn for stroke in Africa. Nat Rev Neurol 17(3):127–128 33452494 10.1038/s41582-021-00456-1

