# Supplementary material for: Revisiting Africa’s Stroke Obstacles and Services (SOS)
Source: Neurol Sci. 2025 Jan 16;46(5):2171–81. doi: 10.1007/s10072-024-07982-y (PMC12003527; doi:10.1007/s10072-024-07982-y)
Supplement: Supplementary file 1 — Supplementary file1 (DOCX 22 KB) [file 10072_2024_7982_MOESM1_ESM.docx]

Supplementary Table 1: Obstacles facing Stroke Services in African Countries based on testimonials from Specialists in the field of Stroke and National Stroke Societies

| Countries | Obstacles facing telestroke service in countries devoid of teleservices | Obstacles facing stroke care in the acute phase | Obstacles facing secondary prevention, post-discharge, and follow-up |
| --- | --- | --- | --- |
| Algeria | Lack of organized communication  Stroke is still not a priority.  Reduced awareness aiding in identifying and managing stroke through teleservices. | Shortness of stroke units, stroke centers, and thrombectomy | Lack of awareness among patients and caregivers about the importance of secondary prevention. |
| Cameroon | Lack of organized communication | No emergency system with a proper stroke management plan  Shortness in stroke specialists  Shortness of stroke units, stroke centers, and thrombectomy | Lack of proper management plan for post-discharge stroke victims.  Financial burden plays a role in losing follow-up visits and losing adherence to medications. |
| DR Congo | Lack of organized communication  Lack of plan to implement teleservices. | Stroke management is not a priority for the government.  Very few governmental hospitals provide stroke services.  Stroke management is expensive, and most Congolese have no health insurance. | Financial burden plays a role in losing follow-up visits and losing adherence to medications. |
| Egypt | - | A lack of awareness makes patients arrive beyond the window.  Lack of organized emergency medical services  Lack of proper communication between health providers and facilities regarding vacant beds | Lack of awareness among patients and caregivers about the importance of secondary prevention. |
| Ethiopia | Patients might arrive with their investigations, so data is not uploaded to the system.  Radiologists and radiology technicians lack knowledge of using the service. | Absence of trained personnel, deficient infrastructure, unavailability of thrombolytic drugs in excess, and thrombectomy  Difficulty accessing imaging modalities and maintaining rapidity of getting images within the proper time window.  Absence of enough dedicated sites for acute stroke care | Financial burden plays a role in losing follow-up visits and losing adherence to medications.  The long-distance needed to reach follow-up clinics and rehabilitation is a major cause of dropouts from follow-up |
| Kenya | Stroke is not a priority.  Lack of funding for such service establishment | Delayed patient presentation  Cost of care as most patients pay out of pocket.  Lack of facilities including dedicated sites for acute stroke care and trained neurologists and nurses | Financial burden plays a role in losing follow-up visits and losing adherence to medications.  Lack of awareness among patients and caregivers about the importance of secondary prevention.  Lack of community strategy and support systems for stroke victims. |
| Libya | Lack of Awareness among patients, relatives, and physicians. | Lack of experience in managing the hyperacute phase of stroke, no neurologist available on hand during the day.  Thrombolytics are not always present (sometimes short in supply), and there is no thrombectomy. | Lack of awareness among patients and caregivers about the importance of secondary prevention.  The low number of rehabilitation facilities and unequal distribution in the country. |
| Mauritius | Not yet a priority for the healthcare services  No expertise and lack of training on how to use it.  Lack of organized communication  High cost of installation | Lack of enough number of neurologists  High cost of acute intervention  Delayed access and lack of coordinated Emergency medical services  Lack of awareness of stroke symptoms | Lack of adherence to control of modifiable risk factors.  Lack of referral from medical doctors for rehabilitation  Lack of access to adapted transport to transfer the patient to the hospital for follow-up (patient often needs a private car or taxi to get to the hospitals and this is very costly)  Patients may get physiotherapy services for free up to 6 months. After 6 months, they can access physiotherapy services privately (out-of-pocket costs).  Lack of awareness by people with stroke and their families on the importance of rehabilitation in the recovery process after a stroke |
| Morocco | Lack of organized communication  Lack of plan to implement teleservices. | A lack of awareness makes patients arrive beyond the window.  Lack of organized emergency medical services  The absence of enough numbers of stroke units and enough trained neuro-interventionists | Absence of dedicated post-stroke consultation and follow-up specialized clinics for stroke victims.  Lack of a sufficient number of rehabilitation centers. |
| Niger | Lack of organized communication | Stroke management is not a priority for the government.  Deficiency in thrombolysis and absence of thrombectomy  Absence of enough number of qualified neurologists and stroke units | Lack of awareness among patients and caregivers about the importance of secondary prevention. |
| Nigeria | Lack of organized communication  Lack of plan to implement teleservices. | A lack of awareness makes patients arrive beyond the window.  Limited access to thrombolysis and thrombectomy with a minimal number of stroke units  Infrastructure constraints in providing timely intervention. | Inadequate management of modifiable risk factors.  Limited access to medications  Healthcare systems focus on acute care rather than long-term management.  Challenges in continuity of care, limited access to rehabilitation services, and lack of standardized protocols for follow-up and monitoring. |
| Seychelles | Lack of organized communication  Lack of plan to implement teleservices | A lack of awareness makes patients arrive beyond the window.  Limited access to thrombolysis and absence of thrombectomy | The Ministry of Health and the government are not prioritizing stroke including care for post-stroke victims, with the absence of neurologists who can take responsibility for post-stroke cases. |
| Somalia | - | Stroke management is not a priority for the government.  A lack of awareness makes patients arrive late.  Lack of financial resources and human capital | Limited public awareness of stroke prevention measures.  Lack of neurologists who prescribe prophylactic medications.  cultural barriers to the importance of follow-up and self-negligence of the patients themselves. |
| South Africa | Lack of organized communication  A limited number of neurologists specifically in the public sector  In 2017 some plans were going yet stalled during the COVID-19 pandemic as health spending was directed fully to the pandemic control | Lack of awareness in the Emergency medical services towards the priority of transferring stroke cases  Lack of priority to prioritize patients with stroke to have brain imaging, attention is focused more on motor car accidents and physical violence which are profound.  Lack of proper training on managing complications related to stroke as aspiration. | Poor risk factor control and adherence to medications.  Underdiagnosis of cardioembolic strokes resulting in poor prescription of anticoagulation.  Inequality in stock and supplies of antihypertensives among hospitals.  The financial burden on caregivers up to losing their jobs secondary to taking care of stroke victims, forces patients to be sent to rural areas to be cared for where lack of facilities is profound. |
| Tanzania | Lack of organized communication | A lack of awareness makes patients arrive beyond the window.  Limited access to thrombolysis and thrombectomy with a minimal number of stroke units | Lack of awareness among patients and caregivers about the importance of secondary prevention. |
| Tunisia | Lack of legal framework to clarify the responsibility of caregivers. | lack of neurologists  poor economic status and financial aspects with associated low resource settings  lack of awareness by patients  no emergency system with a stroke plan, lack of sufficient stroke units, and centers | Lack of awareness among patients and caregivers about the importance of secondary prevention and medication adherence.  Absence of properly dedicated long and middle stay structures to post-stroke care. |
| Zambia | Absence of neuroimaging and enough qualified neurologists | Late presentations to hospitals  Lack of timely access to neuroimaging and stroke specialists  Underdeveloped emergency medical services to transport patients to hospitals or from a lower-level hospital to a higher-level hospital capable of providing hyperacute therapies.  Cost of hyperacute therapies, and lack of trained personnel for thrombectomy | lack of awareness among patients, caregivers, and general practitioners about the importance of secondary prevention.  Erratic supply of necessary medications  lack of inpatient physiotherapy centers and qualified physiotherapists in the outpatient center.  Lack of occupational therapists, and speech therapists.  Transportation and cost barriers for patients to access the post-discharge physician and rehabilitation care they need. |
